# Effect of 6-week treatment with topical betamethasone dipropionate in patients with symptomatic hand osteoarthritis: A randomized double-blind, placebo-controlled trial

**DOI:** 10.1016/j.ocarto.2023.100382

**Published:** 2023-06-16

**Authors:** Yuanyuan Wang, Mahnuma Mahfuz Estee, Desmond Gan, Yuan Z. Lim, Stephane Heritier, Anita E. Wluka, Sultana Monira Hussain, Natalie L. Trevaskis, Flavia M. Cicuttini

**Affiliations:** a School of Public Health and Preventive Medicine, Monash University, Melbourne, VIC 3004, Australia; b Department of Dermatology, Alfred Hospital, Melbourne, VIC 3004, Australia; c Drug Delivery, Disposition and Dynamics, Monash Institute of Pharmaceutical Sciences, Monash University, Parkville, VIC 3052, Australia

**Keywords:** Topical corticosteroid, Osteoarthritis, Hand, Pain, Function

## Abstract

**Objective:**

To examine the efficacy and safety of topical corticosteroid over 6 weeks in patients with symptomatic hand osteoarthritis.

**Design:**

In a randomized, double-blind, placebo-controlled trial, community-based participants with hand osteoarthritis were randomly assigned (1:1) to topical Diprosone OV (betamethasone dipropionate 0.5 ​mg/g in optimised vehicle, n=54) or placebo (plain paraffin, n=52) ointment to painful joints 3 times daily for 6 weeks. Primary outcome was pain reduction [assessed by 100 ​mm visual analogue scale (VAS)] at 6 weeks. Secondary outcomes included changes in pain and function using the Australian Canadian Osteoarthritis Hand Index (AUSCAN), Functional Index for Hand Osteoarthritis (FIHOA), and Michigan Hand Outcomes Questionnaire (MHQ) at 6 weeks. Adverse events were recorded.

**Results:**

Of 106 participants (mean age 64.2 years, 85.9% female), 103 (97.2%) completed the study. Change in VAS at 6 weeks was similar in the Diprosone OV and placebo groups (−19.9 vs. −20.9, adjusted difference 0.6, 95% CI -8.9 to 10.2). There were no significant between-group differences in change in AUSCAN pain [adjusted difference 25.8 (−16.0 to 67.5)], AUSCAN function [21.2 (−55.0 to 97.4)], FIHOA [-0.1 (−1.7 to 1.5)], or MHQ [-1.2 (−6.0 to 3.6)]. Incidence of adverse events was 16.7% in Diprosone OV and 19.2% in placebo group.

**Conclusions:**

Topical Diprosone OV ointment, although well-tolerated, was no better than placebo in improving pain or function over 6 weeks in patient with symptomatic hand osteoarthritis. Future studies should consider examining joints with synovitis and whether delivery approaches enhancing transdermal penetration of corticosteroids into joints are effective in hand osteoarthritis.

**Trial registration:**

ACTRN 12620000599976. Registered May 22, 2020.

## 1 Introduction

Hand osteoarthritis (OA) is a prevalent disabling condition, causing pain, stiffness, reduced hand mobility, and impaired quality of life [[1], [2], [3]]. Currently there are no approved disease-modifying drugs for hand OA to slow disease progression. Clinical practice guidelines recommend topical therapy (non-steroidal anti-inflammatory drugs (NSAIDs), capsaicin), oral analgesics (paracetamol and NSAIDs), chondroitin sulfate, and non-pharmacological therapy (exercises, education, assistive devices, and orthoses) [4,5] for the management of hand OA, mainly focusing on reducing pain and improving function. However, the effect of these therapeutic approaches has been shown to be moderate at best, and associated with significant safety issues [6,7]. There is an unmet need for new improved treatments for hand OA.

Hand OA is a heterogeneous disease with multiple phenotypes, and local inflammation plays a role in causing pain and disease progression [1]. Approximately 50% of patients with hand OA have synovitis [8,9], and the presence of synovitis is associated with joint pain [8,10,11]. These data suggest that inflammation is a treatment target in hand OA, and therapies targeting local inflammation may provide a new approach for the management of hand OA. Corticosteroids are potent anti-inflammatory medications commonly used in rheumatic diseases [12]. Our systematic review and meta-analysis of randomized controlled trials found an effect of oral corticosteroids on reducing pain and improving function over 4–6 weeks in patients with hand OA [13]. Although some clinical trials have shown a beneficial effect of intra-articular corticosteroids in hand OA [14,15], our meta-analysis showed no significant effect of intra-articular injections of corticosteroids [13]. The significant heterogeneity in the drug, frequency, duration and dosage of corticosteroids, the intra-articular treatment for carpometacarpal vs interphalangeal OA, and the use of an active placebo in the intra-articular corticosteroid studies may provide some explanation for the absence of significant effect of intra-articular corticosteroids [13]. However, oral corticosteroids are associated with significant adverse effects including diabetes and osteoporosis [16,17]. Intra-articular injection to hand joints is technically difficult so often needs ultrasound guidance which adds to the cost and inconvenience and affects timeliness of treatment.

Topical application of corticosteroids could provide an alternative approach to reduce local inflammation and improve pain and function in hand OA, by providing more direct delivery of corticosteroid to the joint and minimizing systemic corticosteroid exposure thus reducing the risk of adverse events. Only a few studies have examined the efficacy of topical corticosteroids for arthritis, showing inconsistent results [[18], [19], [20]]. Use of different corticosteroids and delivery methods may have led to differences in efficacy across these studies. The type and potency of the corticosteroid, dose and frequency of administration, and delivery site and method, will all impact the amount of corticosteroid that penetrates into the joint, and thus the efficacy of the treatment after topical delivery [20,21]. Treatment of small joints in the hand with transdermal delivery of corticosteroids is likely to be more achievable than delivery to larger joints where a greater depth of penetration is required to reach into the joint synovium.

We therefore conducted a randomized double-blind, placebo-controlled trial to examine the efficacy and safety of 6 weeks treatment with topical Diprosone OV (Optimised Vehicle) ointment in patients with symptomatic hand OA. Diprosone OV ointment contains the active ingredient betamethasone dipropionate 0.5 ​mg/g. It is used to decrease inflammation, erythema, pruritus and discomfort of many skin conditions, such as eczema, psoriasis and a variety of dermatitis. We chose betamethasone dipropionate because it is highly permeable across the skin relative to other corticosteroids and is the most potent topical corticosteroid approved by the Therapeutic Goods and Administration (TGA) in Australia. The optimised vehicle with the propylene glycol component further increases skin penetration of betamethasone dipropionate and enhances the local effectiveness of the topical corticosteroid. We hypothesised that (1) topical Diprosone OV ointment would be more effective than placebo in reducing pain and improving function in hand OA; and (2) topical Diprosone OV ointment would be safe and tolerable in patients with symptomatic hand OA over 6 weeks.

## 2 Methods

### 2.1 Study design, setting and participants

The current study was a single centre, parallel group, randomized, placebo-controlled, double-blinded trial undertaken between May 25, 2020 and May 18, 2022 in Melbourne Australia. It was registered with the Australian New Zealand Clinical Trials Registry (ACTRN12620000599976, registered May 22, 2020) before recruitment. The study was approved by the Alfred Hospital Ethics Committee (117/20) and the Monash University Human Research Ethics Committee (24,219). Written informed consent was provided by all the participants. The study protocol is published [22].

Participants with hand OA were recruited from the community in Melbourne, Australia via advertisements through local and social media, and from medical practitioners. Inclusion and exclusion criteria are detailed in the published protocol [22]. Inclusion criteria: Participants aged ≥40 years with symptomatic hand OA were recruited. A pain score of ≥40 on a 100 ​mm visual analogue scale (VAS) and radiologic OA (Kellgren and Lawrence grade ≥2) in ≥1 hand joint were required, based on the Osteoarthritis Research Society International (OARSI) clinical trials guidelines for hand OA [23]. Other inclusion criteria included stable analgesic use (including NSAIDs) for at least 4 weeks, stable doses of chondroitin or glucosamine for 4 months, and no corticosteroids via any route for at least 3 months. Exclusion criteria: (i) concomitant rheumatic disease, inflammatory joint disease, psoriatic arthritis, ankylosing spondylitis, or gout; (ii) allergic reaction to medicines containing betamethasone dipropionate, any other corticosteroids, or any of the ingredients of Diprosone OV ointment; (iii) contraindication to topical corticosteroids (e.g. untreated bacterial, fungal, or viral skin lesions; widespread plaque psoriasis; skin conditions with ulcers); (iv) unable to complete informed consent; (v) pregnancy, breast feeding, or trying to become pregnant.

### 2.2 Randomization and masking

Participants were randomly assigned in a 1:1 ratio to self-apply either topical corticosteroid or placebo ointment based on computer generated random numbers prepared and securely held by the Alfred Health Clinical Trials Pharmacy. Block randomization was performed, stratified by gender given the higher prevalence of hand OA in women compared with men [24]. Allocation concealment was ensured by: study medication being dispensed by the Alfred Health Clinical Trials Pharmacy; use of an identical placebo ointment; data collection being taken by assessors blinded to group allocation. Participants, assessors and statisticians were blinded to group allocation.

### 2.3 Intervention

All participants were provided usual care by their health practitioners. Participants in the corticosteroid group applied Diprosone OV ointment (Merck). Participants in the placebo group applied plain paraffin ointment, which had the same colour and appearance as the Diprosone OV ointment. All participants were required to self-apply a thin layer of the study ointment to cover the painful hand joints 3 times per day for 6 weeks.

### 2.4 Study procedure

Volunteers were screened via telephone and underwent an x-ray (standardized posteroanterior view) of the symptomatic hand to confirm the presence of radiologic disease. If bilateral symptomatic hands were present, the most symptomatic hand underwent x-ray. If bilateral symptomatic hands had the same pain level, the dominant hand underwent x-ray. The study visits were performed via telehealth. Consent was obtained by the study doctor. At baseline, all the hand joints with a pain level of ≥40 on a 100 ​mm VAS were documented on a hand map. The participants were provided a copy of the marked hand map for reference when they were followed up by study staff via telephone. Participants were asked to apply the study ointment on each of these pre-specified hand joints 3 times per day for 6 weeks. Participants were allowed to continue the treatments being taken at the screening and baseline visit during the trial. Participants were able to withdraw at any time during the trial, with the time and reasons being recorded. They were asked to complete the questionnaires at 6 weeks. Study data were collected and managed using REDCap electronic data capture tools hosted and managed by Helix (Monash University) [25].

### 2.5 Endpoints

The primary endpoint was reduction in hand VAS pain at 6 weeks, according to the OARSI recommendations for the design and conduct of clinical trials for hand OA [23]. Participants were asked to rate pain in their hand joints over the last 7 days using a 100 ​mm VAS at baseline and 6 weeks. VAS has been most frequently used for pain assessment in hand OA, showing excellent reliability, good construct validity and sensitivity to change [26].

Secondary endpoints were change in pain and function at 6 weeks, assessed using the Australian Canadian Osteoarthritis Hand Index (AUSCAN) pain (0–500), stiffness (0–100), and function (0–900) subscales [27], Functional Index for Hand OA (FIHOA) (0–30) [28], and Michigan Hand Outcomes Questionnaire (MHQ) (0–100) [29], according to the OARSI recommendations [23]. The overall MHQ score was calculated using the formula [Function ​+ ​Activities of Daily Living ​+ ​Work ​+ ​(100-Pain) ​+ ​Aesthetics ​+ ​Satisfaction]/6. Participants were asked about their pain levels weekly using a numeric rating scale (NRS, 0–10).

### 2.6 Safety

Participants were asked about any adverse events at each follow-up by research staff. Details of the adverse event and its relationship with the intervention were recorded. Hand photos (back and front) were taken at baseline and 6 weeks and reviewed by a dermatologist who was blinded to group allocation for safety check, mainly looking for dilatation of vessels, skin wrinkling, and hypopigmentation. Photos at 3 weeks were also taken and reviewed if there was any concern.

### 2.7 Other measures

Success of participant blinding was assessed, where participants were asked which treatment they believe they received. The ointment container was weighed before and after the study to assess treatment adherence. Central sensitization was assessed using painDETECT, a validated questionnaire used to assess neuropathic pain in OA [30].

### 2.8 Sample size calculation

To detect a 15 ​mm between-group difference in VAS pain change at 6 weeks (primary outcome), which is the minimal clinically important difference to be detected in OA trials [31], with a standard deviation of 20 ​mm for VAS change [32], alpha 0.05 and two-sided significance, 40 participants per group were required to attain a power of 90%. Accounting for an estimated 20% loss to follow-up, we sought to recruit 100 participants, 50 in each group.

### 2.9 Statistical analysis

The statistical analysis plan is described in the study protocol [22]. The primary analyses were based on intention-to-treat analysis of both the primary and secondary outcomes including all the participants as allocated in their randomized groups regardless of their adherence to assigned treatments. The primary endpoint was reduction in hand pain at 6 weeks on a 100-mm VAS, and the secondary endpoints were change in AUSCAN, FIHOA, and MHQ scores at 6 weeks. The differences in these endpoints between the treatment groups were assessed using Analysis of Covariance (ANCOVA) with adjustment for the baseline measure. Differences between randomized groups in trajectories of pain over time were examined using linear mixed-effects regression models with baseline score as the covariate, fixed factors for treatment, time, and treatment ​× ​time interaction, and with an autoregressive AR(1) covariance matrix for repeated measures of individuals over time. Analyses were undertaken based on the pre-specified subgroups of joint group, radiographic severity, pain level, and presence of central sensitization. The statistical significance was based on a p-value of <0.05 (two-sided). Stata 16.0 (StataCorp LP., College Station, TX, USA) was used for the statistical analyses.

## 3 Results

**Fig. 1:**
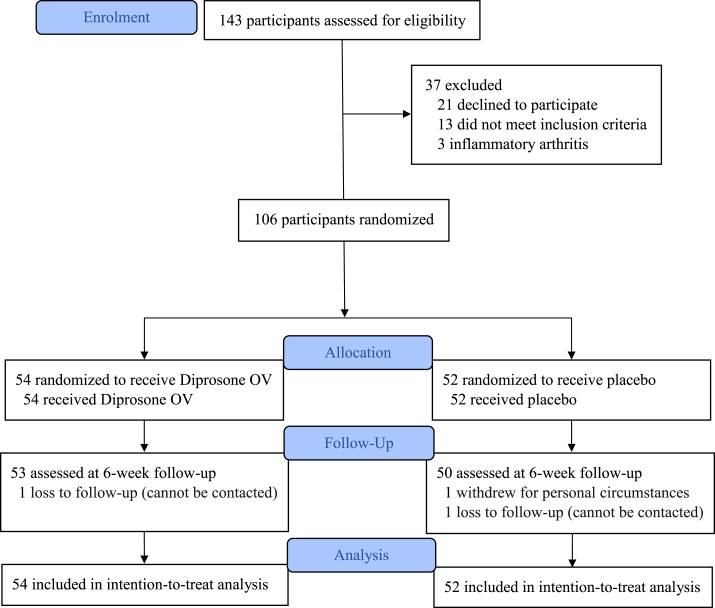
Study flowchart.

From May 25, 2020 to February 4, 2022, 143 participants were screened and 106 were randomized to apply either Diprosone OV (n=54) or placebo (n=52) ointment (Fig. 1). Characteristics of study participants are presented in Table 1. Mean age was 64.2 [standard deviation (SD) 7.4] years, and 91 (85.9%) were female. Baseline characteristics were generally well balanced between the Diprosone OV and placebo groups. One hundred and three (97.2%) participants completed the study, and three (2.8%) withdrew [1 (1.9%) in the Diprosone OV group and 2 (3.8%) in the placebo group. Weighing of returned study medication demonstrated 88.1% of the participants complying with study medication of >80%, 90.2% in the Diprosone OV group and 86.0% in the placebo group.

**Table 1:** Baseline characteristics of study participants.

	Diprosone OV (N=54)	Placebo (N=52)
Age, years	63.0 (6.8)	65.4 (7.9)
Female, n (%)	46 (85.2)	45 (86.5)
Body mass index (n=53/49), kg/m^2^	27.2 (4.4)	26.4 (3.7)
Disease duration, years	4.5 (3.7)	4.3 (4.4)
Kellgren Lawrence grade^a^ (n=51/51), n (%)		
Grade 1	1 (2.0)	1 (2.0)
Grade 2	18 (35.3)	22 (43.1)
Grade 3	14 (27.4)	8 (15.7)
Grade 4	18 (35.3)	20 (39.2)
Number of painful joints in study hand	5.7 (4.6)	5.4 (4.8)
Painful CMC joint, n (%)	44 (81.5)	31 (59.6)
Painful DIP joints, n (%)	33 (61.1)	30 (57.7)
Painful PIP joints, n (%)	29 (53.7)	32 (61.5)
Concomitant medications, n (%)		
Acetaminophen	31 (57.4)	21 (40.4)
Oral NSAIDs	15 (27.8)	22 (42.3)
Topical NSAIDs	6 (11.1)	4 (7.7)
Fish oil	7 (13.0)	9 (17.3)
Turmeric/curcumin	6 (11.1)	9 (17.3)
Glucosamine/chondroitin	6 (11.1)	6 (11.5)
Other analgesics	5 (9.3)	4 (7.7)
VAS (0–100)	61.4 (20.0)	62.3 (18.9)
AUSCAN pain (0–500)	265.7 (123.4)	247.2 (112.3)
AUSCAN stiffness (0–100)	52.4 (26.1)	46.2 (30.2)
AUSCAN function (0–900)	467.4 (235.0)	441.0 (236.4)
FIHOA (0–30) (n=52/50)	10.1 (5.8)	8.9 (6.3)
MHQ (0–100) (n=52/51)	54.8 (16.9)	57.2 (15.1)

Data presented as mean (SD) or n (%).

CMC: carpometacarpal joint; DIP: distal interphalangeal joints; PIP: proximal interphalangeal joints; NSAID: nonsteroidal anti-inflammatory drug; VAS: visual analogue scale; AUSCAN: Australian Canadian Osteoarthritis Hand Index; FIHOA: Functional Index of Hand Osteoarthritis; MHQ: Michigan Hand Outcomes Questionnaire.

a The Kellgren Lawrence grade of the study hand was the maximum Kellgren Lawrence grade among the CMS, DIP and PIP joints.

### 3.1 Primary and secondary endpoints

At 6 weeks, a reduction of VAS pain was observed in both Diprosone OV (−19.9, SD 26.1) and placebo (−20.9, SD 26.5) groups, with no significant between-group difference (adjusted difference 0.6, 95% confidence interval (CI) −8.9 to 10.2) (Table 2).

**Table 2:** Primary and secondary outcomes at 6 weeks.

	Baseline Mean (SD)		Change between baseline and week 6 Mean (SD)		Between group difference Mean (95% CI)^a^	P^a^
	Diprosone OV (N=54)	Placebo (N=52)	Diprosone OV (N=54)	Placebo (N=52)		
**Primary endpoint**						
VAS	61.4 (20.0)	62.3 (18.9)	−19.9 (26.1)	−20.9 (26.5)	0.6 (−8.9, 10.2)	0.89
**Secondary endpoints**						
AUSCAN pain	265.7 (123.4)	247.2 (112.3)	−63.5 (126.0)	−80.4 (106.3)	25.8 (−16.0, 67.5)	0.22
AUSCAN stiffness	52.4 (26.1)	46.2 (30.2)	−13.6 (23.0)	−11.9 (29.0)	1.6 (−8.0, 11.2)	0.74
AUSCAN function	467.4 (235.0)	441.0 (236.4)	−88.6 (229.9)	−98.0 (192.6)	21.2 (−55.0, 97.4)	0.58
FIHOA	10.1 (5.8)	8.9 (6.3)	−1.0 (4.0)	−0.5 (3.9)	−0.1 (−1.7, 1.5)	0.90
MHQ	54.8 (16.9)	57.2 (15.1)	7.9 (12.4)	8.2 (12.1)	−1.2 (−6.0, 3.6)	0.62

SD: standard deviation; CI: confidence interval; VAS: visual analogue scale; AUSCAN: Australian Canadian Osteoarthritis Hand Index; FIHOA: Functional Index of Hand Osteoarthritis; MHQ: Michigan Hand Outcomes Questionnaire.

a Adjusted for baseline measure.

**Fig. 2:**
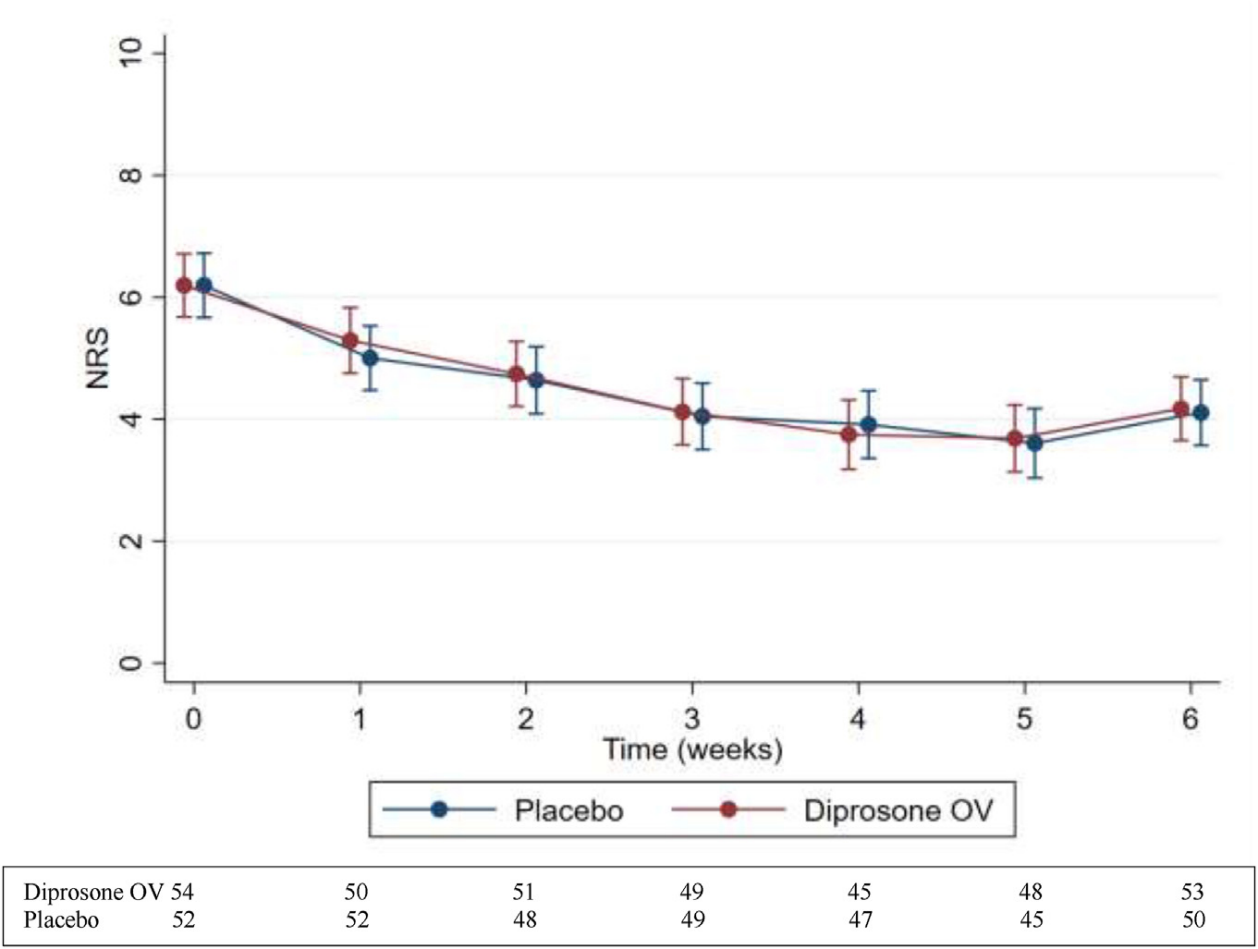
Numeric rating scale of the study hand at each time point over 6 weeks (mean. and 95% confidence interval).

At 6 weeks, both Diprosone OV and placebo groups experienced reduced pain and improved function, demonstrated by decreased AUSCAN pain [−63.5 (SD 126.0) vs. −80.4 (SD 106.3)], AUSCAN stiffness [−13.6 (SD 23.0) vs. −11.9 (SD 29.0)], AUSCAN function [−88.6 (SD 229.9) vs −98.0 (SD 192.6)], and FIHOA [−1.0 (SD 4.0) vs. −0.5 (SD 3.9)] scores, and increased MHQ score [7.9 (SD 12.4) vs 8.2 (SD 12.1)] (Table 2). For all these secondary outcomes, there were no significant differences between the Diprosone OV and placebo groups (Table 2). When weekly pain was examined (Fig. 2), a reduction in pain was observed after 1 week of treatment in both groups, which continued until 5 weeks of treatment. At 6 weeks, pain level was slightly increased compared with the 5-week level in both groups. There were no significant differences observed between the two groups.

### 3.2 Pre-specified subgroup analyses

Subgroup analysis was performed based on the involvement of carpometacarpal joint, distal interphalangeal joints, and proximal interphalangeal joints, respectively (Supplementary Tables 1–3), severity of radiographic OA (Supplementary Table 4), pain level (Supplementary Table 5), and presence of central sensitization (Supplementary Table 6). There were no significant between-group differences in the primary and secondary outcomes, except for greater improvements in AUSCAN pain in the placebo group compared with the Diprosone OV group in participants with distal interphalangeal joint involvement and those with proximal interphalangeal joint involvement.

### 3.3 Adverse events

Adverse events during the study period are presented in Table 3; 13 in the Diprosone OV group and 14 in the placebo group. Nine (16.7%) participants in the Diprosone OV group and 10 (19.2%) in the placebo group reported ≥1 adverse event during the trial. Nearly all of the adverse events were mild (25 out of 27) with only 2 adverse events being moderate (one in each group). No action was taken for the adverse events, except one participant in the Diprosone OV group discontinued study medication and withdrew from the study due to adverse event (rash and redness, steroid allergy). The majority of the adverse events were assessed as possibly related to the study medication, 2 adverse events as unlikely related, and one adverse event as not related. There was no serious adverse event.

**Table 3:** Adverse events.

	Diprosone OV (N=54)	Placebo (N=52)
Number of adverse events	13	14
Number of serious adverse events	0	0
Number (%) of participants with adverse event		
Any adverse event	9 (16.7)	10 (19.2)
Skin colour becoming red/brown/dark	5 (9.3)	3 (5.8)
Itchiness	3 (5.6)	4 (7.7)
Feeling hot or warm	1 (1.9)	2 (3.8)
Rash	2 (3.7)	1 (1.9)
Dryness and/or flakes	0	2 (3.8)
Swelling	0	1 (1.9)
Skin thinning	0	1 (1.9)
Mild headache	1 (1.9)	0
Dermatitis (eczema or irritant dermatitis) on finger tips	1 (1.9)	0

### 3.4 Success of participant blinding

There were no significant differences between the Diprosone OV and placebo groups in terms of their perception of which treatment they received throughout the trial (Table 4).

**Table 4:** Success of participant blinding.

Does the participant believe they are on active or placebo	Diprosone OV	Placebo	p
Week 1			0.84
Active	13 (25.5)	13 (25.0)	
Placebo	16 (31.4)	19 (36.5)	
Not sure	22 (43.1)	20 (38.5)	
Week 2			0.31
Active	13 (25.5)	15 (32.6)	
Placebo	22 (43.1)	13 (28.3)	
Not sure	16 (31.4)	18 (39.1)	
Week 3			0.25
Active	20 (42.6)	19 (39.6)	
Placebo	18 (38.3)	13 (27.1)	
Not sure	9 (19.2)	16 (33.3)	
Week 4			0.13
Active	23 (52.3)	17 (36.2)	
Placebo	15 (34.1)	16 (34.0)	
Not sure	6 (13.6)	14 (29.8)	
Week 5			0.95
Active	21 (45.7)	20 (44.4)	
Placebo	16 (34.8)	17 (37.8)	
Not sure	16 (19.6)	8 (17.8)	
Week 6			0.41
Active	26 (53.1)	19 (39.6)	
Placebo	17 (35.7)	21 (43.8)	
Not sure	6 (12.2)	8 (16.7)	

Data presented as no (%).

## 4 Discussion

Six-week treatment with topical Diprosone OV ointment 3 times daily, although safe and well tolerated, was not more effective than placebo in reducing pain and improving function in participants with symptomatic hand OA. These findings do not support the use of topical Diprosone OV ointment in the treatment of hand OA.

There is evidence from a systematic review and meta-analysis for a medium effect of oral corticosteroid on improving pain and function over 4–6 weeks in hand OA [13]. This current study explored the potential of topical corticosteroid to reduce pain and improve function in hand OA as a way to reduce the well-known systemic complications of oral corticosteroids. However, although we found that topical corticosteroid was safe and well tolerated, we did not find a significant difference in pain and functional improvement between the Diprosone OV ointment and placebo groups, with both groups experiencing significant improvements in pain and function at 6 weeks.

There are a number of explanations for our findings. Both the intervention and placebo groups had very significant improvements in pain and function. Studies in OA have often reported very high placebo effects [33]. This may, in part, reflect the tendency for pain in OA to fluctuate and improve in the medium term. This may be in part due to the natural history of OA, including hand OA, to improve. We recruited participants who had significant symptoms at the time of study inclusion, so it is most likely that what we were capturing in the placebo group is the natural history of hand OA, a return towards the person's baseline. Furthermore, although the placebo ointment had no analgesic component, it is possible that the observed analgesia effect may be attributable to a beneficial effect of rubbing [34]. The heterogeneity of our study population may be another explanation. In the previous trial of oral prednisolone that showed an effect on hand OA [32], study participants with symptomatic hand OA and signs of inflammation were recruited from rheumatology outpatient clinics, including those with ≥4 distal and proximal interphalangeal joints with osteoarthritic nodes, ≥1 distal and proximal interphalangeal joint with soft swelling or erythema, ≥1 distal and proximal interphalangeal joint with a positive power Doppler signal or synovial thickening of at least grade 2 on ultrasound, and finger pain of ≥30 ​mm on a 100-mm VAS that flared up during a 48-h NSAID washout (defined as worsening of finger pain by ​≥ ​20 ​mm on the VAS). This was a more clinically severe patient population with imaging evidence of synovitis in contrast to our community-based population with symptomatic hand OA with no requirement for the presence of synovitis. It may be that future studies should focus on patients with hand OA and joints with evidence of synovitis as these would be most likely to benefit from topical corticosteroids, rather than joints where the synovitis has resolved and residual symptoms are due to the degenerative changes.

Topical Diprosone OV ointment was safe and generally well tolerated. The most common adverse events were skin reactions, occurring in 14.8% (8/54) of the Diprosone OV group. However, placebo group utilising plain paraffin ointment (not usually known to cause skin reaction) also reported adverse skin reaction of 19.2% (10/52). Most of the adverse events were mild with no action needed, and there was no serious adverse event. Only one participant withdrew due to adverse event. All patients accepted the treatment, and 90.2% of participants in the Diprosone OV group complied with the study medication of >80%.

Our study has limitations. The Diprosone OV is designed for localised treatment of skin conditions and not designed for transdermal penetration into the joint. The dose of corticosteroid penetrating into the joint is also unknown, as is the dose required. Thus, it is possible that we did not deliver enough drug into the joints. There are strengths in our study. Successful participant blinding was evidenced in our study. There was also high compliance with the treatments and high follow-up rate.

Given the lack of effective therapies for hand OA, there is the opportunity to build on the findings from the previous clinical trials of oral corticosteroids and our current findings. It will be important to focus any further testing on those with evidence of synovitis as this population is most likely to benefit. We chose to test the efficacy of treatment with Diprosone OV ointment as this represents the most potent corticosteroid ointment currently on the market. This preparation contains both the potent corticosteroid betamethasone diproprionate 0.5 ​mg/g and the optimised vehicle containing propylene glycol to enhance penetration of the corticosteroid into the skin [21,35]. In our study, patients applied the ointment three times daily in an attempt to maintain the highest possible concentrations of the corticosteroid in the joint throughout the treatment period since a previous study showed no effect of corticosteroids applied twice weekly with iontophoresis or phonophoresis in treatment of patients with trapeziometacarpal arthritis [20]. There were, however, limitations to our study design in that the dose and concentration of betamethasone diproprionate required to treat hand OA are not well defined. In a vasoconstrictor test, the minimum concentration at which betamethasone diproprionate is active is 0.000016% which is an extremely low concentration [35]. However, the direct applicability of this test to the clinical situation is uncertain. For the treatment of a range of conditions, including various forms of arthritis, betamethasone diproprionate is given at a dose of 0.5–9 ​mg via intravenous, intramuscular or oral routes. Previous studies have found percutaneous absorption of up to 14% of the dose for betamethasone diproprionate administered on the back skin of healthy adults [36]. In our study, absorption of betamethasone diproprionate would have been enhanced by the optimised vehicle, and thinness of the skin barrier over the hand joints. Nonetheless, there was limited therapeutic benefit from the Diprosone OV ointment which may relate to delivery of an inadequate amount of the corticosteroid to the joint since oral corticosteroids are an effective treatment for hand OA. Previous clinical trials have shown an effect of topical NSAIDs (diclofenac sodium gel) in OA [37,38], suggesting the negative results of the current study may be due to the difficulties or inadequate corticosteroid absorbed through the skin to the joint. However, as discussed previously, it may also be that the heterogeneity of our patient population may have impacted any benefit. Future studies can focus on optimising the transdermal delivery of corticosteroids to hand joints with evidence of synovitis using novel delivery approaches such as penetration enhancing formulations, dressings, patches, and iontophoresis. These studies will need to encompass further investigation into the dose-response relationships for corticosteroids in the joint in the setting of hand OA and synovitis.

In conclusion, self-applied topical Diprosone OV ointment 3 times daily, although generally well tolerated, was no better than placebo in improving pain or function over 6 weeks in community-based patients with symptomatic hand OA. Future studies should consider examining joints with evidence of synovitis and novel delivery approaches to enhance transdermal penetration of corticosteroids to the joint that may improve outcomes for hand OA.

## Role of the funding source

The study is funded by an Investigator Grant from the National Health and Medical Research Council of Australia (NHMRC, APP1194829), a Project Grant from the Arthritis Australia, and a Monash University Joint Medicine Pharmacy Grant. YW is the recipient of NHMRC Translating Research into Practice Fellowship (APP1168185). MME is the recipient of Bangabandhu Science and Technology Fellowship from Ministry of Science and Technology, Government of the People's Republic of Bangladesh. YZL is the recipient of NHMRC Clinical Postgraduate Scholarship (APP1133903) and Royal Australasian College of Physicians Woolcock Scholarship. AEW is the recipient of the Royal Australian College of Physicians Fellows Career Development Fellowship. SMH is the recipient of NHMRC Early Career Fellowship (APP1142198). FMC is the recipient of NHMRC Investigator Grant (APP1194829). The funder of the study had no role in the study design and conduct of the study; collection, management, analysis and interpretation of the data; preparation, review, or approval of the manuscript; and decision to submit the manuscript for publication.

## Credit author statement

Yuanyuan Wang: Conceptualization, Data curation, Formal analysis, Funding acquisition, Investigation, Methodology, Project administration, Resources, Supervision, Roles/Writing – original draft, Writing – review & editing.

Mahnuma Mahfuz Estee: Data curation, Investigation, Writing – review & editing.

Desmond Gan: Conceptualization, Data curation, Investigation, Methodology, Writing – review & editing.

Yuan Z. Lim: Data curation, Investigation, Writing – review & editing.

Stephane Heritier: Data curation, Formal analysis, Methodology, Writing – review & editing.

Anita E. Wluka: Data curation, Funding acquisition, Investigation, Methodology, Writing – review & editing.

Sultana Monira Hussain: Conceptualization, Funding acquisition, Methodology, Writing – review & editing.

Natalie L. Trevaskis: Conceptualization, Funding acquisition, Methodology, Writing – review & editing.

Flavia M. Cicuttini: Conceptualization, Data curation, Formal analysis, Funding acquisition, Investigation, Methodology, Project administration, Resources, Supervision, Roles/Writing – original draft, Writing – review & editing.

The final manuscript has been read and approved by all the authors. Yuanyuan Wang and Flavia M. Cicuttini had full access to all the data in the study, took responsibility for the integrity of the data and the accuracy of the data analysis, and had final responsibility for the decision to submit for publication.

## Declaration of competing interest

The authors declare that they have no conflict of interest.
