# Supplementary material for: Effect of 6-week treatment with topical betamethasone dipropionate in patients with symptomatic hand osteoarthritis: A randomized double-blind, placebo-controlled trial
Source: Osteoarthr Cartil Open. 2023 Jun 16;5(3):100382. doi: 10.1016/j.ocarto.2023.100382 (PMC10329169; doi:10.1016/j.ocarto.2023.100382)
Supplement: Multimedia component 1 [file mmc1.pdf]

Supplementary Table 1: Primary and secondary outcomes at 6 weeks in participants with carpometacarpal joint involvement

	Baseline Mean (SD)		Change between baseline and week 6 Mean (SD)		Between group difference Mean (95% CI)*	P*
	Diprosone OV (N=44)	Placebo (N=31)	Diprosone OV (N=44)	Placebo (N=31)		
**Primary endpoint**						
VAS	61.5 (3.0)	65.5 (3.5)	-21.3 (26.3)	-16.6 (30.7)	-6.9 (-19.2, 5.4)	0.26
**Secondary endpoints**						
AUSCAN pain	269.3 (18.8)	266.1 (22.1)	-61.5 (132.7)	-70.5 (123.2)	9.8 (-46.1, 65.8)	0.73
AUSCAN stiffness	51.6 (4.1)	52.8 (5.9)	-13.2 (24.9)	-12.8 (34.4)	0.1 (-12.6, 12.7)	0.99
AUSCAN function	483.8 (36.5)	505.9 (40.4)	-88.3 (243.8)	-96.8 (218.2)	-1.8 (-104.7, 101.0)	0.97
FIHOA	10.4 (0.9)	9.9 (1.3)	-1.0 (4.2)	-0.1 (3.8)	-0.7 (-2.6, 1.3)	0.51
MHQ	54.2 (17.1)	53.9 (15.0)	8.0 (12.0)	7.1 (13.8)	0.8 (-5.4, 6.9)	0.81

SD: standard deviation; CI: confidence interval

*Adjusted for baseline measure

Supplementary Table 2: Primary and secondary outcomes at 6 weeks in participants with distal interphalangeal joint involvement

	Baseline Mean (SD)		Change between baseline and week 6 Mean (SD)		Between group difference Mean (95% CI)*	P*
	Diprosone OV (N=33)	Placebo (N=30)	Diprosone OV (N=33)	Placebo (N=30)		
**Primary endpoint**						
VAS	65.8 (3.4)	67.7 (2.9)	-16.6 (23.1)	-24.4 (22.1)	7.1 (-4.0, 18.1)	0.21
**Secondary endpoints**						
AUSCAN pain	277.0 (21.7)	272.5 (18.2)	-27.5 (98.4)	-81.8 (104.7)	**56.3 (4.8, 107.7)**	**0.03**
AUSCAN stiffness	59.8 (4.4)	54.2 (5.4)	-10.2 (20.1)	-12.3 (29.5)	5.3 (-7.4, 18.0)	0.41
AUSCAN function	477.1 (41.8)	520.2 (40.6)	-15.9 (174.1)	-109.7 (175.1)	83.1 (-4.4, 170.5)	0.06
FIHOA	10.4 (1.1)	10.2 (1.0)	-0.4 (3.2)	0.04 (3.9)	-0.3 (-2.2, 1.6)	0.77
MHQ	51.2 (17.4)	53.1 (14.3)	5.9 (9.7)	9.6 (12.5)	-4.4 (-10.0, 1.1)	0.11

SD: standard deviation; CI: confidence interval
*Adjusted for baseline measure

Supplementary Table 3: Primary and secondary outcomes at 6 weeks in participants with proximal interphalangeal joint involvement

	Baseline Mean (SD)		Change between baseline and week 6 Mean (SD)		Between group difference Mean (95% CI)*	P*
	Diprosone OV (N=29)	Placebo (N=32)	Diprosone OV (N=29)	Placebo (N=32)		
**Primary endpoint**						
VAS	68.0 (3.5)	67.3 (2.9)	-19.5 (24.2)	-26.6 (23.8)	7.5 (-4.4, 19.5)	0.21
**Secondary endpoints**						
AUSCAN pain	282.1 (24.1)	265.5 (19.5)	-29.3 (100.9)	-83.5 (118.6)	**58.8 (4.3, 113.4)**	**0.04**
AUSCAN stiffness	62.5 (4.3)	54.7 (5.2)	-12.8 (19.9)	-13.6 (32.0)	4.9 (-8.8, 18.6)	0.48
AUSCAN function	465.2 (47.0)	497.6 (43.5)	-2.0 (180.9)	-106.2 (228.8)	88.0 (-12.3, 188.3)	0.08
FIHOA	10.7 (1.2)	10.8 (1.2)	-0.6 (3.2)	-0.4 (4.1)	-0.05 (-2.0, 1.9)	0.96
MHQ	50.7 (18.4)	52.3 (15.2)	5.9 (10.6)	10.0 (12.3)	-4.7 (-10.3, 1.0)	0.11

SD: standard deviation; CI: confidence interval

*Adjusted for baseline measure

Supplementary Table 4: Primary and secondary outcomes at 6 weeks stratified by the severity of radiographic hand osteoarthritis

	Baseline Mean (SD)		Change between baseline and week 6 Mean (SD)		Between group difference Mean (95% CI)*	P*	P for interaction*
	Diprosone OV (N=51)	Placebo (N=51)	Diprosone OV (N=51)	Placebo (N=51)			
**Primary endpoint**							
VAS							
K-L grade 1-3	62.3 (21.4)	60.4 (16.8)	-20.0 (23.9)	-20.1 (20.0)	0.9 (-10.2, 11.9)	0.88	0.74
K-L grade 4	63.0 (17.9)	65.4 (22.2)	-22.3 (29.4)	-21.6 (34.9)	-2.8 (-21.4, 15.8)	0.76	
**Secondary endpoints**							
AUSCAN pain							
K-L grade 1-3	283.3 (119.5)	249.5 (105.3)	-79.4 (125.8)	-73.1 (88.0)	6.7 (-48.2, 61.6)	0.81	0.25
K-L grade 4	252.9 (128.3)	240.9 (127.0)	-24.2 (126.1)	-85.0 (129.9)	64.1 (-5.4, 133.7)	0.07	
AUSCAN stiffness							
K-L grade 1-3	54.1 (27.8)	47.1 (27.9)	-15.9 (25.4)	-10.2 (20.4)	-3.0 (-14.7, 8.8)	0.61	0.29
K-L grade 4	50.2 (25.6)	44.1 (34.6)	-7.1 (17.9)	-13.6 (39.1)	10.1 (-7.9, 28.1)	0.26	
AUSCAN function							
K-L grade 1-3	484.8 (234.9)	434.8 (223.0)	-126.7 (229.0)	-114.6 (134.5)	8.7 (-84.4, 101.8)	0.85	0.47
K-L grade 4	459.9 (244.0)	444.4 (265.7)	9.3 (214.8)	-62.9 (254.3)	71.0 (-64.1, 206.2)	0.29	
FIHOA							
K-L grade 1-3	10.0 (5.2)	7.7 (5.6)	-1.2 (4.6)	-0.1 (3.6)	-0.6 (-2.9, 1.6)	0.58	0.38
K-L grade 4	11.0 (6.7)	10.8 (7.1)	-0.6 (3.4)	-1.2 (4.5)	1.2 (-1.3, 3.6)	0.34	
MHQ							
K-L grade 1-3	56.2 (16.8)	58.5 (14.7)	7.2 (12.3)	8.2 (11.8)	-1.8 (-8.1, 4.4)	0.56	0.83
K-L grade 4	48.9 (15.8)	54.8 (16.1)	9.4 (11.1)	8.6 (13.2)	-1.0 (-9.3, 7.3)	0.81	

SD: standard deviation; CI: confidence interval; K-L: Kellgren Lawrence
*Adjusted for baseline measure

Supplementary Table 5: Primary and secondary outcomes at 6 weeks stratified by pain level

	Baseline Mean (SD)		Change between baseline and week 6 Mean (SD)		Between group difference Mean (95% CI)*	P*	P for interaction*
	Diprosone OV (N=54)	Placebo (N=52)	Diprosone OV (N=54)	Placebo (N=52)			
**Primary endpoint**							
VAS							
VAS <70	47.4 (13.4)	49.0 (13.8)	-12.6 (22.7)	-15.5 (26.1)	1.8 (-10.4, 14.0)	0.76	0.79
VAS ≥70	80.3 (8.3)	79.1 (7.3)	-29.4 (27.6)	-27.7 (26.0)	-0.7 (-16.5, 15.2)	0.93	
**Secondary endpoints**							
AUSCAN pain							
VAS <70	208.5 (129.7)	192.3 (93.1)	-45.1 (130.1)	-69.0 (73.6)	29.9 (-19.2, 79.1)	0.23	0.87
VAS ≥70	342.8 (53.8)	316.3 (96.1)	-86.0 (119.7)	-95.0 (137.9)	25.7 (-48.4, 99.7)	0.49	
AUSCAN stiffness							
VAS <70	42.8 (25.5)	36.0 (26.9)	-9.5 (22.6)	-9.6 (21.6)	2.9 (-8.4, 14.2)	0.61	0.66
VAS ≥70	65.3 (21.4)	59.0 (29.7)	-18.7 (23.1)	-14.9 (36.7)	0.1 (-16.9, 17.1)	0.99	
AUSCAN function							
VAS <70	398.9 (251.6)	335.7 (214.6)	-76.2 (248.5)	-79.6 (133.4)	25.4 (-74.0, 124.8)	0.61	0.81
VAS ≥70	559.7 (176.6)	573.8 (194.6)	-103.6 (209.6)	-121.4 (250.3)	10.0 (-111.3, 131.3)	0.87	
FIHOA							
VAS <70	9.8 (6.2)	6.1 (4.5)	-2.0 (4.4)	-0.7 (2.5)	-0.2 (-2.0, 1.7)	0.86	0.76
VAS ≥70	10.5 (5.3)	12.1 (6.7)	0.3 (3.0)	-0.1 (5.5)	0.3 (-2.5, 3.0)	0.84	
MHQ							
VAS <70	60.8 (14.4)	64.3 (12.6)	7.8 (12.7)	9.4 (9.5)	-2.4 (-8.4, 3.7)	0.43	0.52
VAS ≥70	46.6 (16.8)	48.6 (13.6)	8.0 (12.4)	6.6 (14.9)	-0.1 (-7.6, 7.3)	0.97	

SD: standard deviation; CI: confidence interval

*Adjusted for baseline measure

Supplementary Table 6: Primary and secondary outcomes at 6 weeks stratified by the presence of central sensitization

	Baseline Mean (SD)		Change between baseline and week 6 Mean (SD)		Between group difference Mean (95% CI)*	P*	P for interaction*
	Diprosone OV (N=36)	Placebo (N=39)	Diprosone OV (N=36)	Placebo (N=39)			
**Primary endpoint**							
VAS							
painDETECT score <13	59.0 (18.1)	57.5 (19.8)	-22.3 (26.5)	-15.5 (26.0)	-6.0 (-19.7, 7.7)	0.38	0.19
painDETECT score ≥13	71.0 (18.6)	75.2 (11.1)	-17.4 (21.9)	-30.8 (30.9)	11.6 (-13.5, 36.7)	0.34	
**Secondary endpoints**							
AUSCAN pain							
painDETECT score <13	234.0 (126.6)	234.0 (117.9)	-61.4 (127.1)	-77.4 (83.4)	19.6 (-33.9, 73.1)	0.47	0.63
painDETECT score ≥13	327.9 (82.7)	293.4 (35.9)	-45.1 (135.9)	-74.6 (181.5)	60.3 (-75.1, 195.7)	0.36	
AUSCAN stiffness							
painDETECT score <13	39.8 (26.3)	39.6 (27.2)	-12.4 (24.0)	-10.5 (25.2)	-0.5 (-12.8, 11.8)	0.93	0.43
painDETECT score ≥13	67.9 (24.8)	70.6 (28.0)	-9.7 (18.4)	-19.6 (40.8)	9.6 (-19.9, 39.2)	0.50	
AUSCAN function							
painDETECT score <13	398.4 (232.7)	385.5 (231.1)	-90.7 (239.3)	-83.5 (149.9)	6.5 (-94.4, 107.4)	0.90	0.94
painDETECT score ≥13	548.5 (231.3)	600.7 (230.8)	-64.8 (215.1)	-105.3 (318.1)	7.6 (-212.1, 226.4)	0.95	
FIHOA							
painDETECT score <13	8.8 (5.0)	6.4 (4.8)	-1.5 (4.2)	-0.8 (3.4)	-1.9 (-4.2, 0.5)	0.12	0.25
painDETECT score ≥13	12.4 (7.1)	14.0 (7.7)	-1.0 (3.6)	-1.9 (5.5)	0.9 (-3.7, 5.4)	0.69	
MHQ							
painDETECT score <13	59.5 (15.1)	62.8 (12.9)	7.7 (13.2)	6.4 (9.8)	0.2 (-6.3, 6.7)	0.96	0.90
painDETECT score ≥13	47.2 (22.1)	46.5 (14.5)	6.8 (7.8)	7.3 (21.1)	-0.6 (-16.5, 15.3)	0.93	

SD: standard deviation; CI: confidence interval

*Adjusted for baseline measure
